# 1940. Real-Life Impact of Pneumococcal Conjugate Vaccines (PCVs) on Hospitalization of Young Children with RSV-Associated Community-acquired Alveolar Pneumonia (CAAP)

**DOI:** 10.1093/ofid/ofad500.094

**Published:** 2023-11-27

**Authors:** Ron Dagan, Bart Adriaan van der Beek, David Greenberg, Shalom Ben-Shimol

**Affiliations:** Ben-Gurion University of the Negev, Beer Sheva, HaDarom, Israel; Ben-Gurion University of the Negev, Beer Sheva, HaDarom, Israel; Soroka University Medical Center, Pediatric Infectious Disease Unit, Beer Sheva, HaDarom, Israel; Soroka University Medical Center, Beer Sheva, HaDarom, Israel

## Abstract

**Background:**

The important role of *streptococcus pneumoniae* in pediatric community-acquired alveolar pneumonia (CAAP) has been demonstrated, mainly through PCV impact on CAAP rates in young children, in whom RSV is also frequently detected. The causative role of RSV on CAAP has been strongly suggested from multiple epidemiological studies. Although pneumococcal-RSV coinfections in pneumonia were suggested by a pre-licensee PCV9 study, no solid real-life data were generated to substantiate the potential effect of PCVs on RSV-associated CAAP (RSV-CAAP). The current prospective study evaluates the impact of PCV7/PCV13 implementation in Israel on all-cause CAAP and RSV-CAAP incidence dynamics in young children during RSV seasons.

**Methods:**

PCV7/PCV13 were implemented in Israel in July 2009/November 2010. A prospective population-based surveillance on CAAP rates in children < 5 years in southern Israel has been ongoing since 2002 (Ben-Shimol, CID 71:177, 2020). Nasal samples of RSV (by PCR) were tested in most episodes and were extrapolated for total CAAP episodes. RSV season was defined as all months with >5 positive RSV tests in southern Israel. The impact was determined by incidence rate ratio (IRR) comparing each RSV season (and the four years 2015-19 combined) to the five pre-PCV implementation years, 2004-09, combined).

**Results:**

From Jul-2004 to Jun-2019, 7,654 CAAP episodes were recorded; 3,661 (47.8%) were tested for RSV, of which 1,662 (45.4%) were positive. The seasonal proportions of RSV-CAAP of all episodes ranged from 37.4% to 67.2%. Compared to 2004-09 (combined), IRR (95% CI) for 2015-19 were 0.68 (0.56-0.84) for RSV CAAP and 0.70 (0.61 – 0.80) for all-cause CAAP (**Table, Figure**). Generally, yearly seasonal IRRs for all-cause CAAP and RSV-CAAP were similar.

**Figure:**
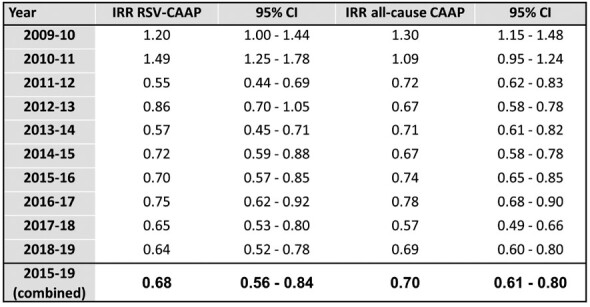

**Figure:**
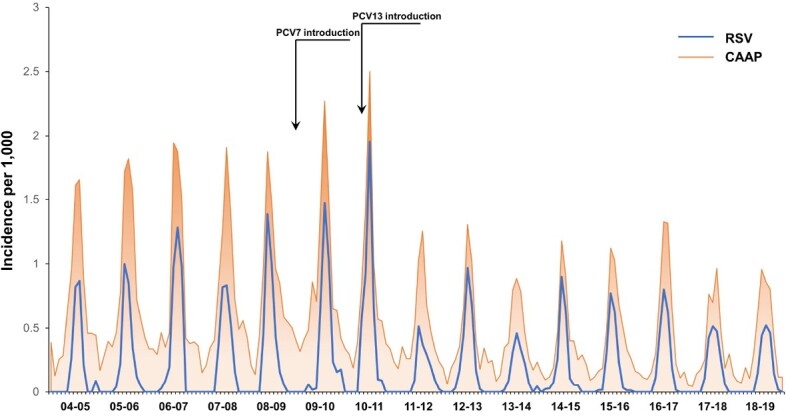

**Conclusion:**

The abrupt, steep , and seasonal reduction of hospitalization of young children for both all-cause CAAP and RSV-CAAP strongly support the important role of RSV-*S. pneumoniae* coinfections in CAAP. The dynamics observed in CAAP and other pneumococcus-associated diseases in association with RSV activities during COVID-19 (Dagan, EBioMedicne, 90:104993, 2023) together with the real-life impact of PCVs on RSV-CAAP confirm the mutual role of RSV and *S. pneumoniae* in CAAP.

**Disclosures:**

**Ron Dagan, Professor MD**, GSK: Honoraria|MedIMmune/AstraZeneca: Grant/Research Support|MSD: Advisor/Consultant|MSD: Grant/Research Support|MSD: Honoraria|Pfizer: Advisor/Consultant|Pfizer: Expert Testimony|Pfizer: Grant/Research Support|Pfizer: Honoraria|Sanofi Pasteur: Honoraria **David Greenberg, Professor MD**, GSK: Advisor/Consultant|GSK: Honoraria|MSD: Advisor/Consultant|MSD: Grant/Research Support|MSD: Honoraria|Pfizer: Advisor/Consultant|Pfizer: Honoraria **Shalom Ben-Shimol, Dr. MD**, GSK: Honoraria|MSD: Advisor/Consultant|MSD: Honoraria|Pfizer: Advisor/Consultant|Pfizer: Grant/Research Support|Pfizer: Honoraria

